# How group singing facilitates recovery from the symptoms of postnatal depression: a comparative qualitative study

**DOI:** 10.1186/s40359-018-0253-0

**Published:** 2018-08-17

**Authors:** Rosie Perkins, Sarah Yorke, Daisy Fancourt

**Affiliations:** 1 Centre for Performance Science, Royal College of Music, Prince Consort Road, London, SW7 2BS UK; 2 Faculty of Medicine, Imperial College London, London, SW7 2AZ UK; 3 Present Address: Faculty of Medicine, Imperial College London, London, SW7 2AZ UK; 4 Present Address: Department of Behavioural Science and Health, University College London, London, WC1E 7HB UK

**Keywords:** Postnatal depression, Singing, Community, Music, Qualitative, Focus groups

## Abstract

**Background:**

Previous research has demonstrated that making music can enhance positive emotions as well as support positive psychological functioning. However, studies tend to be limited by lack of comparison with other psychosocial interventions. This study builds on a three-arm randomised controlled trial (RCT) that demonstrated that group singing for mothers and babies, but not group creative play, can lead to faster recovery from moderate-severe symptoms of postnatal depression than usual care. The aim was to elucidate the mechanisms of the group singing intervention in order to account for its recovery properties.

**Methods:**

Qualitative research was conducted with 54 mothers who had experienced symptoms of postnatal depression. Mothers completed a 10-week programme of either group singing or group creative play as part of the wider RCT study. Data were collected via a series of 10 semi-structured focus groups conducted at the end of each 10-week programme. These were designed to elicit subjective and constructed experiences of the singing and play interventions and were analysed inductively for emergent themes.

**Results:**

Five distinctive features of the group singing emerged: (i) providing an authentic, social and multicultural creative experience, (ii) ability to calm babies; (iii) providing immersive ‘me time’ for mothers; (iv) facilitating a sense of achievement and identity; (v) enhancing mother-infant bond.

**Conclusions:**

Community group singing interventions may reduce symptoms of postnatal depression through facilitating a functional emotional response rooted in the needs of new motherhood. These features are of relevance to others seeking to implement creative interventions for maternal mental health.

**Trial registration:**

NCT02526407. Registered 18 August 2015.

## Background

Postnatal depression (PND) is a debilitating condition characterised by fatigue and low energy, insomnia and anhedonia. Prevalence figures vary, but it is estimated that PND affects 12.9% of mothers with at least 75,000 cases per year in the UK alone [1, 2]. The impact of PND can be severe, with suicide being a leading cause of maternal death in the first year after childbirth [2], and indications that PND can impact negatively on children’s cognitive, socio-emotional and behavioural development [3–5]. Additionally, PND can continue to impact on how mothers parent their children after the postpartum period [6], and maternal PND can impact negatively on fathers’ experiences of parenting [7].

Consequently, there has been significant attention given to the best treatment models for PND. Boath & Henshaw identified five main treatment areas: pharmacological; psychological and psychodynamic; pharmacological and psychological; hormonal; and social support and relaxation [1]. In a 2013 systematic review, Dennis & Dowswell demonstrated that psychosocial and psychological interventions significantly reduce the number of women who develop PND, with professionally-based home visits, telephone peer support and interpersonal psychotherapy showing promise [8]. Focusing on social support, Boath & Henshaw reviewed a number of social interventions including support groups, massage therapy and relaxation. While there were methodological flaws in the impact evidence provided, their recommendation was for further research to be conducted into a field that may well prove fruitful [1]. Subsequently, Honey, Bennett, & Morgan reported a controlled psycho-educational group intervention that reduced scores on the Edinburgh Postnatal Depression Scale (EPDS), but led to no changes in perceptions of social support, coping or marital relationships [9]. Creative social interventions have also received attention. Perry, Thurston, & Osborn reported that a creative arts group was felt by mothers to be a relaxed safe space [10] while Feeley, Bell, Hayton, Zelkowitz, & Carrier showed that mothers perceived creative activities to provide social interaction and relaxation, decrease monotony and meet other personal needs [11].

Nonetheless, there remain a lack of psychosocial creative intervention studies for women with PND. This is perhaps surprising given the rapidly accumulating evidence base for the positive role of the arts in mental health and wellbeing [12–14]. Crawford, Lewis, Brown, & Manning (2013) argued that the arts have an important place in mental health recovery, with the potential to facilitate spaces of compassion, trust and shared understanding [15]. Indeed, studies from music [16–19], dance [20, 21] and art [22, 23] have all contributed to evidence that creative arts activities can support and enhance aspects of mental health. Singing, in particular, has received research attention in relation to its health benefits. Among the general public, singing has been demonstrated to be a joyful, life-enhancing activity that promotes wellbeing [24]. Among participants experiencing mental distress, singing has been shown to aid recovery from serious or enduring mental illness [25], and to facilitate personal and social impact as well as functional outcomes for adults living with a chronic mental illness or disability [26]. Further, von Lob, Camic, & Clift demonstrated that group singing may be a useful coping strategy for people living through times of adversity [27]. Finally, Kreutz demonstrated that choral singing, but not chatting, can lead to increases in oxytocin, a hormone associated with social bonding [28], a point reinforced by other studies [29, 30].

Given the prevalence and severity of PND, and challenges with current treatment models such as low compliance or lack of availability of suitably trained professionals [2], new psychosocial community interventions that build upon the existing evidence from arts and health are timely. In particular, the potential for singing to enhance bonding and to facilitate personal, social and functional impact may directly address the psychosocial risk factors connected with PND [31]. In order to explore this, a randomised controlled trial (RCT) was carried out [32]. This compared the effects of a 10-week programme of group singing for new mothers and their babies with 10 weeks of usual care. While previous studies have attempted to explain the mechanisms behind music’s impact on health [33, 34], these are limited by a lack of comparison with other, perhaps equally effective, psychosocial interventions. Consequently, in order to start to identify whether group singing per se is an effective intervention or whether other group social engagement is equally effective, a third arm was included within the RCT comprising 10 weeks of group creative play activities for mothers with symptoms of PND and their babies.

The RCT revealed that symptoms of PND reduced significantly faster amongst mothers in the singing group with moderate-severe PND than for mothers in the usual care group. Interestingly, there was no significant difference in recovery between the singing and play groups, as measured by EPDS scores at baseline, week 6 and week 10, or between the play and control groups [32]. This suggests that group singing may have specific benefits over and above the comparison psychosocial intervention. Yet why this happened, and the mechanisms underpinning the singing group, remain unknown. As DeNora and Ansdell argued, RCTs run the risk of ‘the middle period, in other words the time in which music is active, [being] left in shadow, which means that the processes by which music might be “having an effect” are left in darkness, made mysterious’ ([35], no page number). Indeed, that qualitative methods can inform and/or illuminate the results of an RCT is also acknowledged within wider mental health research [36, 37]. Therefore, this study aimed to explore *how* the group singing programme facilitated recovery from the symptoms of PND through a qualitative analysis of the experiences of mothers involved in the singing group and comparisons with qualitative data from mothers in the play group. The driving research question was: What are the specific features of a community group singing intervention known to support recovery from symptoms of PND?

## Methods

### Procedure

#### The interventions

The RCT on which this study is based had three arms: singing (experimental), creative play (comparison) and usual care (control). RCT participants randomised to the singing and play groups received free 60 min workshops for them and their baby every week for 10 weeks in a community children’s centre. Between 8 and 12 women plus their babies were recruited into each group, and five singing groups and five play groups ran over a six-month period in 2016 (*n* = 134 women completed the RCT and provided full data). Groups were led by one of two professional workshop leaders, specially recruited to work on the project and with experience of facilitating music and/or creative workshops in community settings. The leaders were supported by a team of seven specially-trained conservatoire students (one per each 10-week programme) and a project coordinator. The two leaders led both singing and creative play sessions to ensure consistency between the two conditions, and worked together to plan materials and content. Singing workshops involved mothers listening to songs sung by the leader, learning and singing songs with their babies and creating new songs together reflecting aspects of motherhood. Creative play workshops involved mothers engaging in sensory play with their babies, doing arts and crafts and playing simple games together. Songs and creative play activities were selected initially by the workshop leader, but were also suggested by the participants. They were designed to be engaging for the mothers as well as to support the mothers interacting with their babies (e.g. through singing lullabies or through designing art work based on the babies’ handprints). Participants provided background and demographic data as well as completing the EPDS at baseline, week 6 and week 10. The median number of sessions attended by women was eight for the singing group and six for the play group. Full details of the RCT intervention and outcomes are published elsewhere [32, 38].

#### Methodology and methods of data collection

The study was underpinned by social constructionism, assuming that the interventions were socially constructed and that the ways in which mothers reported their salient features represented socially constructed knowledge. The qualitative design therefore sought a rich understanding of the features of the singing groups as they were reported by the participants themselves. In an attempt to isolate the specific features of the singing group that might have led to a faster reduction in PND symptoms, this study qualitatively compared the experiences of mothers in the singing group with the experiences of mothers in the play group.

Data were collected via a series of 10 focus groups, divided equally between five focus groups for singing and five focus groups for play. Focus groups were selected to account for and capture the shared experiences and understandings constructed within each group over the 10-week period, although in one case there was only one woman in attendance and so this was conducted as a semi-structured interview. Focus groups were held immediately or soon after the final session of each 10-week programme, and women self-selected to attend. The schedule concentrated on experiences of the intervention and of new motherhood. Each focus group was facilitated by one researcher, comprised on average 5.4 members (see Table 1), and lasted for between 16 and 26 min. The women attended with their babies and therefore it was not appropriate to aim for a lengthy discussion, so the facilitator aimed to draw out the salient points as efficiently as possible. This point may also account for the low uptake in some groups, as attending data collection with a baby included significant logistical challenges. The focus groups were audio recorded with permission and fully transcribed.

**Table 1:** Participant characteristics, organised by focus group

Focus Group	Number of participants	Age range of mothers (years)	Mean number of weeks post-birth	Mean EPDS score^1^	Educated to degree level^2^ (%)	Household income above £61 k (%)	With a partner (%)
Sing1	9						
Sing2	7						
Sing3	7	22–43	19.36	14.51	88.57%	65.63%	91.43%
Sing4	5						
Sing5	9						
Play1	2						
Play2	4						
Play3	2	31–45	17.13	13.50	87.50%	75.00%	87.50%
Play4	8						
Play5	1^3^						

Note 1: EPDS ≥10 indicative of possible symptoms of PND. EPDS ≥13 indicative of moderate-severe symptoms of PND

Note 2: Missing data points excluded from all % calculations

Note 3: Run as a semi-structured interview

#### Participants

Participants included in the RCT were women with babies up to 40 weeks post birth who scored 10 or higher on the EPDS at baseline, indicating some symptoms of PND. Women were recruited through midwives, doctors, perinatal psychiatrists, health visitors and General Practitioners (GPs) in the Greater London area of the United Kingdom (UK), as well as through social media, leaflets and by a project coordinator in children’s centres and in the local community. Women were excluded from the RCT if their baby was outside the specified age range (0–9 months), if a healthcare professional advised that the intervention was not suitable for them (in practice we recorded no instances of this), if they did not or could not provide informed consent or if they lived outside the Greater London area. Women were not expected to have any prior experience or knowledge of singing. The UK National Health Service (NHS) South East Scotland Research Ethics Committee approved the project [reference 15/SS/016], and women gave written informed consent.

All women who had participated in either the singing or play groups as part of the wider RCT study were invited to take part in this study through email and oral communication at the sessions, and 54 women volunteered and consented to take part. Of these, four did not provide data for the RCT study, meaning that of the 91 women who completed the singing and play workshops in the RCT, 50 (55%) are represented in this study. Participant characteristics are presented in Table 1; across the 54 participants, 92% were first time mothers. Nationalities represented in the sample included British, French, Polish, Canadian, Columbian, Australian, Japanese, Italian and North American.

#### Analysis

An inductive thematic analysis of the transcripts was undertaken, acknowledging that there is as yet no one established theory as to music’s effects on mental health. The analysis proceeded in four main steps, specifically designed to provide a qualitative description [39] of the constructed features of the singing intervention. First, each transcript was read for familiarity before, second, important units of meaning were selected and labelled as emergent nodes in NVivo10 by the first author. The units of meaning emerged inductively, but there was a pre-determined focus on the features of the singing/play activities constructed by the participants as important or meaningful. Third, the units of meaning were cross-checked by the second author and additions or changes were discussed until consensus was reached. Finally, the units of meaning were clustered into sub-themes and, ultimately, overarching themes that characterise the main features of the singing and play activities, again cross-checked between two researchers. Sub-themes were only classed as such when they were represented across at least three of the five respective focus groups. Reflexivity was addressed through the interplay between the two analysts, both of whom had a different position in relation to the research and the participants: one as the lead researcher, only in attendance at a small number of the sessions and the second as the project coordinator, in attendance at the vast majority of sessions over the course of the RCT and the first point of contact for the women. These different positions enabled both analysts to engage with the data from their own starting points, to recognise where different interpretations may lie and to agree a shared understanding of the central themes.

## Results

Four overarching themes identified the main features of the two interventions: (1) activity mechanisms (features of the activity itself), (2) environmental mechanisms (features of the environment created within the sessions), (3) social mechanisms (social features of the activity), and (4) psycho-emotional mechanisms (psychological and/or emotional features of the activity). A total of 13 sub-themes emerged for the singing activity (labelled S) and a total of 9 sub-themes emerged for the play activity (labelled P), as summarised in Table 2.

**Table 2:** Overarching themes and sub-themes

Themes^1^	Sing sub-themes	Play sub-themes
1. Activity mechanisms	S1.1 ‘Authentic’ musical engagement S1.2 New singing skills S1.3 Singing outside of class	P1.1 Varied play activities P1.2 New play ideas P1.3 Playing outside of class
2.Psycho-emotional mechanisms	S2.1 Singing feels good S2.2 Singing time for mums S2.3 Singing as immersive S2.4 Singing as achievement and purpose S2.5 Singing supports bonding	P2.1 Play feels good
3. Social mechanisms	S3.1 Singing impact on babies (calming) S3.2 Singing as part of group S3.3 Singing supports routine	P3.1 Playing as part of group P3.2 Play supports routine P3.3 Play impact on babies
4. Environmental mechanisms	S4.1 Calm and inclusive singing environment S4.2 Importance of singing leader	P4.1 Calm and inclusive play environment P4.2 Importance of play leader

Note 1: Overarching themes are organised in terms of qualitative strength for the singing group. Sub-themes are organised into qualitative strength for the singing and play groups respectively

In what follows, each overarching theme will be described in turn. Sub-themes from the singing activity will be presented alongside sub-themes from the play activity, either to demonstrate consistent features across the two activities or to illustrate differences. Indicative quotations are used to support each sub-theme, but interpretation is reserved for the following discussion.

### Activity mechanisms

Two activity sub-themes were consistent across both singing and play. First, both groups reported that they took away new skills or ideas from the activities (sub-themes ‘new singing skills’ and ‘new play ideas’):

I’ve found it very rewarding to have something to take away with me each week as well. Coming in to being a mum, and knowing a few songs, but not many, it’s been really nice to learn songs [Sing 3].

I find it’s hard to imagine what to do creatively at home with him and now we’ve got lots of really creative ideas and I feel really inspired [Play 4].

Additionally, the play group reported that they appreciated the variety of activities introduced across the programme, and the flexibility with which the leaders introduced activities to meet the needs of the group (sub-theme ‘varied play activities’):

I think the nice thing about this group is that there is a structure, but it’s been changing based on the feeling of the day of the group and I think that is the best kind of group. You have some kind of structure, but then it’s very kind of – what’s the word – flexible [Play 1].

Second, across both groups, the activities learned were reported to be transferable to other contexts (sub-themes ‘singing outside of class’ and ‘playing outside of class’), whether at home or in other, sometimes challenging, circumstances:

It gives good ideas, when you go home and you’re like ‘oh I can play with this or make that’ [Play 2].

Just this weekend we were back in the hospital and she was having to have blood taken and she was going mental, and I found myself singing the [folk lullaby] song to her and it just gave me something that I might not have ... I don’t know, a nursery rhyme or something, I might not have thought to do that, but something about the repetitiveness and the fact that we’ve done it lots of times. It made a difference to have that [Sing 3].

One further feature emerged from the singing group, which was seen as a form of ‘authentic’ musical engagement (sub-theme ‘authentic musical engagement’). Participants felt that the groups were natural and calming rather than commercial, with singing that drew upon diverse influences and music contributed both by the leader and the participants:

I’ve been to some other music classes and things like that and I’ve found them, really really cheesy and almost like sensory overload by the end of it, and I like how [leader] really pays attention to reading all the babies and calming things down when she needs to and livening it up when they’re ready for it and stuff like that, and that it doesn’t feel like commercial and cheesy, it feels very authentic, lovely music that you can sing at home and not feel like a cartoon character or something [Sing 2].

I love that they were quite global songs, like some are Indonesian. That’s just a wonderful thing. To be in a group of women singing global songs was quite powerful I thought, so that was nice. Not just nursery rhymes [Sing 4].

It’s been really nice to learn songs from different cultures and know that you don’t necessarily need to know what they mean, and they don’t need to be English words. It’s just really lovely to know different songs. You can use them with a little bit more amusing music to calm [baby] and entertain her [Sing 3].

Indeed, singing was seen as particularly beneficial when it was ‘multimodal’, or presented in parallel with another activity or resource:

[It] was really nice because it was combined - ‘I’ll read her a book; I’ll sing her a song’. I never really thought to do that; to actually bring those things together was really nice [Sing 3].

To summarise, both groups felt that they learned new activities to do with their babies and reported increased confidence in doing so, as well as transferability outside of the sessions. The mothers in the singing group appreciated the authentic nature of the musical engagement, and particularly the multicultural experience as well as the use of other creative forms (such as stories) to accompany the singing.

### Psycho-emotional mechanisms

Across both groups, the activities were perceived as enhancing positive emotions (sub-themes ‘singing feels good’ and ‘play feels good’):

It’s one of the activities that I look forward to and we will certainly miss not coming to the sessions any more (…) You feel kind of uplifted and pleased that you came [Play 1].

It’s very uplifting. I leave here a lot happier than I started [Sing 2].

Indeed, the activities were seen as particularly uplifting or supporting in the context of a challenging time in new motherhood:

No matter how bad the night you’ve had, no matter how knackered you are, you’ve got to still just get out and go to the group. Because it just makes you feel better, don’t you think? [Sing 1].

I think parts of this course has actually helped me get through the sort of darker elements of ... the darker days of when it does feel endless, and when it does feel tough….because it’s [the singing] a fixed thing, it’s something I’ve got to get out of the house for, and it’s something that I know that even if it’s crappy to get here, actually once I’m here I know that it will be nice and it will be a relaxed atmosphere. Whether she’s crying, or whether she’s hungry, or whether she’s sleeping, or whether she’s playing, all of that is actually nice, and accepted, and fine [Play 5].

Despite this transversal experience, however, it was in this theme of psycho-emotional mechanisms that the most striking differences emerged between the singing and the play groups.

First, women in the singing group perceived the session as a time for mothers, and not only an activity designed for their babies (sub-theme ‘singing time for mums’):

Everything is for the baby. You go to a class and it’s always for the baby. Then you go out and meet for coffee with your friends and you talk about your babies. This [singing] is also good for the baby, but at the same time it’s something for us as well [Sing 3].

Perhaps as a result of this perceived focus, the singing sessions also emerged as a form of ‘me time’, where the mothers could do an activity for themselves:

I hadn’t really thought about music really, helping me. Especially at the beginning, you’re just surviving, I think. But as soon as I started singing, it seemed to relax me and really made a big difference to [my baby] [Sing 4].

I think even though you’re actively participating it almost does feel like a bit of down time as well. It’s a bit of relaxing time. *Another woman*: Yeah, that’s why it’s nice for mums as well [Sing 3].

Linked with these points, the singing sessions were also reported to be immersive for some of the mothers (sub-theme ‘singing as immersive’):

Because I’m still working, I work throughout, I’m always using my mobile phone and it’s the one time that I actually have never picked up my phone. Normally in a class I’ll just check my phone, I’ll just check my emails, but actually I haven’t in this class. So that’s been hugely beneficial to me, just to have that time out that I don’t normally give myself [Sing 2].

I think it’s so helpful just being here, like being in the present, instead of thinking about what am I going to do in five minutes. I need to prep this, prep that (…) Here, I’m just being here singing, and that’s a huge difference [Sing 5].

Interestingly, some the features of the singing itself seem to be instrumental to this immersion, facilitating a musical experience that can also be aesthetically absorbing:

With the singing I find that, I said before about how it’s nice all the songs having a beginning and an end, and I find the way [the leader] would sometimes make it fast, sometimes slow, sometimes loud, sometimes quiet, that’s something really lovely about getting lost in the song in that way [Sing 2].

Moving on, the singing also enabled a sense of achievement (sub-theme ‘singing as achievement and purpose’):

Sometimes it makes me anxious that you are doing all that is expected, taking care of your baby, but other than that you are doing nothing (…) So in this sense as well, I think coming to here and I started to sing ... I feel like I was doing at least something in a more tangible way. I was coming here, I did something today [Sing 4].

We went through a period of her not being very well, but with breastfeeding not working, and her losing weight that was quite stressful. Coming to this made me feel like I was doing something that was really nurturing her while I felt like I was struggling to nurture her, so that really made a difference in that time [Sing 3].

We see here the women reflecting on the daily challenges that they faced as new mothers, and the ways in which singing helped them to discover a sense of accomplishment. Singing also appeared to support a reconnection with a sense of self and purpose that had been lost in the transition to motherhood:

*Speaker 1*: When I first came (…) I wasn’t very well, and as a result, was slightly lacking in confidence and I think over a period of time, as I’ve gotten better and sort of been on medication as well as coming to the class and as well as interacting with lovely people - mothers, again - it’s been hugely beneficial to me, I feel. I feel like new again and I think in a big way in the beginning that wasn’t there. I never thought I would feel like me again, post baby. *Speaker 2*: You came back to yourself again. *Speaker 1*: Yes [Sing 2].

I think just feeling, like you go from work, which is another identity, and then you go off a bit lost into motherhood identity, where you know your role but you probably don’t know what it means. Having songs, and having this, it helps you to be able to add to your purpose, and you’ve got some strings to your bow. We’re all great mums, but sometimes you feel like you’re not. You need, if you come to something like this, you feel like you’ve got different songs and things [Sing 3].

Finally, a small number of women reported that singing helped them to bond with their baby (sub-theme ‘singing supports bonding’):

It helps the bond between us, too. Something that [the leader] first said, on the first day was actually that they want to hear the sound of your voice, so don’t be scared about singing [Sing 3].

I have a good relationship with him because I sing to him every day, some songs - so it’s useful [Sing 5].

To summarise, mothers in both groups reported that their activity facilitated positive emotions, especially in relation to the challenges of new motherhood. Among the singing group only, the mothers also reflected on singing being a form of ‘me time’; a space for them as well as for their baby that could also be immersive. Additionally, singing facilitated a sense of achievement for the mothers, particularly in relation to nurturing their babies and rediscovering a sense of self and purpose. Finally, for some of the mothers singing was a means of enhancing the mother-baby bond.

### Social mechanisms

Both sets of mothers reported that they benefited from being part of a group (sub-themes ‘singing as part of group’ and ‘playing as part of group’):

I felt like I was really part of the group (…) with so many of the other drop in classes and groups and things, you don’t really get to know the babies that well and you’re more focused on you and the baby and what’s going on, not like you and everyone else and their babies (…) So it’s been really nice to feel part of a group [Play 2].

You feel part of the community, and (…) it’s lovely [Sing 4].

In addition to the experience of being a group member, this social forum also provided a means of learning from other mothers:

I think you learn from other mums as well. You see how they are with their children (…) so it’s not only just about the music, it’s about like you get to interact with other mums and see how they parent their babies and I might implement like that in my routine with my child and so it’s a lot more than just the music [Sing 2].

You can learn a lot from each other (…) just how much you can learn off each other in really informal way [Play 1].

That the mothers are brought together regularly each week appears to create an opportunity for sharing tips and resources that are beneficial to their ongoing experiences of motherhood. Finally, among the singing group this social cohesion seems to be strengthened through the act of singing:

I think if it was shorter, if it was only two or three sessions, it wouldn’t really work. I think the first time I came, I came away saying ‘Oh, that’s nice, but it’s nothing that you wouldn’t get from another group or something’ but then after ten weeks, you really do feel like the songs become the group [Sing 3].

Singing as a group, that’s one of the things that I like [Sing 2].

While all participants experienced the benefits of feeling part of a regular group and learning from other mothers, those in the singing group also benefited from the social cohesion facilitated by the music itself.

Furthermore, both singing and play participants described their activities as a motivation and structure for getting out of the house with a young baby (sub-themes ‘singing supports routine’ and ‘play supports routine’):

I had a difficult labour and first few months keeping the baby healthy, and I was home a lot with the challenges and this gave me an opportunity to meet other people, so that I’m not alone with kids [Play 4].

I think it was really good for me to have something to go to, that meant I wasn’t home all the time. It was also something different, away from just walking to [the] park again [Sing 3].

In addition to facilitating a change of scene, the activities also provided routine within the mothers’ week:

I always have something to do each day. And knowing that we have something to come to [Play 4].

I didn’t have ideas of how to start or how to start again, reorganise my daily life and to have routine. So having the session every week, every Friday at the same time, actually I think it helps to re-establish your rhythm [Sing 4].

Additionally, the mothers reported that they enjoyed seeing the impact of both singing and play activities on their babies (sub-themes ‘singing impact on babies’ and ‘play impact on babies’):

It’s just the way that [the babies] are with each other and you can see that they remember each other (…) it just shows that once a week and with the music and the drum and then the singing, they’ve all developed and they’ve all grown up so much [Sing 2].

Seeing the babies develop and change week on week, in a kind of creative setting, has been really lovely [Play 2].

For mothers in all the singing groups, it was also reported that singing calmed their babies, including outside of the sessions:

The songs have (…) been calming for [baby] when he’s really crying. Just standing like this, singing some of the songs. Sometimes you just get into a zone where you’re just singing them on repeat in a trance singing it … [Sing 1].

I can use them. Lots of times, like in the middle of the night, I sing hours on end. Rocking him [Sing 5].

Socially, then, the activities provided a sense of group belonging for the participants, in which knowledge about motherhood could also be shared. For the singing group in particular, this sense of social cohesion was strengthened by the act of singing itself. Both activities provided a sense of structure and routine in the mothers’ lives and a shared experience of seeing their babies develop. Finally, singing was also reported as a useful way to help calm and soothe babies.

### Environmental mechanisms

For mothers in both groups, the environment in the sessions was reported to be calm (sub-themes ‘calm and inclusive singing environment’ and ‘calm and inclusive play environment’):

I think it was also a really calm environment. That was good [Play 3].

It’s much calmer than any of the other baby classes that we go to together [Sing 2].

Furthermore, the mothers experienced the spaces as non-judgemental, particularly in relation to their babies’ behaviour:

I knew that nobody would mind if she [baby] was squawking and people have been really supportive about her wanting to be carried around, ok let’s take it in turns to do that (...) I remember when I started this group, I was still slightly in the phase of I would go to things and be slightly on edge about whether she’d be in a meltdown, and I can feel that I’ve relaxed. Not necessarily in every setting, I wouldn’t like it if it happened on the train or something, but here, yes, I know that it’s absolutely fine and it will be okay, and I think that’s helped my confidence with her as well, so that’s been really nice [Play 5].

I think it’s been nice about this group – it’s because wherever you go, as a mum, you feel judged. But here, it’s just like naked. We’re all in exactly the same position [Sing 1].

Linked with this, the mothers appeared to appreciate the lack of pressure put on them to participate in the activities in a certain way or to a certain degree:

That it, there’s no pressure to get super involved [Play 2].

I don’t sing very well at all, but we don’t judge each other here and it’s just all about just being isn’t it [Sing 1].

In the play group, some of the mothers also recognised the value of the sessions as an opportunity to talk – alongside the creative play activities – in a trusting space:

It felt a little more comfortable here to discuss certain things and quite open conversations, and it’s all felt very comfortable and very trusting, warm environment [Play 1].

Moving to the final sub-theme, both groups emphasised the vital importance of the leader in facilitating effective interventions for new mothers (sub-themes 'importance of singing leader’ and ‘importance of play leader’):

I think [leader] was a really lovely moderator, and I think the atmosphere that she created was very relaxed and very positive. So a big element is actually having her as the person who’s brought it all together [Play 5].

[Leader] brings her experience and positivity. I think she brought a lot of humanity into it, that’s probably why we really liked it and the kids reacted that way [Sing 1].

In sum, the calm and inclusive leader was identified as important to the mothers, as was the quality and temperament of the workshop leaders.

## Discussion

This article has scrutinised the features of a community group singing intervention known to support recovery for mothers with moderate-severe symptoms of PND. By situating data from the singing group alongside data from women in a parallel creative play group, the aim has been to illuminate the *specific* features of the singing group. Nonetheless, across both interventions a series of transversal mechanisms emerged to account for the potential benefits of more generic creative interventions for postnatal mental health recovery: (1) a shared experience for mothers of seeing babies develop and enjoy a creative activity; (2) learning new activities to do with babies, and increased confidence in doing this outside of the intervention sessions themselves; (3) an enhanced sense of ‘feeling good’; (4) a sense of group belonging, in which knowledge about motherhood can be shared; (5) a sense of structure and routine in daily life; (6) a calm and inclusive environment, facilitated by high quality creative leaders and support team. Given that low levels of social support are widely acknowledged to predict PND [31], it is particularly interesting that many of the social benefits of being part of a creative group were evident across both singing *and* play, perhaps helping to account for the lack of a significant difference in recovery speed found in the RCT between these two conditions [32]. However, as we also know from the RCT, singing – but not play – led to more rapid reductions in moderate-severe symptoms of PND than usual care [32], suggesting that there are other factors that may differentiate the impact of singing.

Indeed, a number of features emerged that may account for this activity’s ability to reduce PND symptoms. Beck includes self-esteem as a predictor of PND based on her meta-analysis of 84 studies [40]. That singing allows women to feel a sense of achievement, specifically in caring for and nurturing their baby, is of relevance here, contributing to what Leahy-Warren, McCarthy, & Corcoran term maternal parental self-efficacy, or ‘mothers’ beliefs about their ability to be successful in the parenting role’ ([41], p.390). Leahy-Warren, McCarthy, and Corcoran’s research posited a link between maternal parental self-efficacy and reduced symptoms of PND, suggesting that the role of singing to enable a mother to feel able to look after and care for her baby may be important. Further, that singing can calm babies may also support this point; with the ‘tool’ of singing to support being able to calm down a crying baby, mothers may feel more competent and able to deal with challenging infant behaviour or situations. Indeed, other studies have pinpointed the importance of postnatal interventions in supporting aspects of maternal confidence [42], although evidence on this is limited [43].

The mothers’ reports of the singing sessions as relaxing and immersive, creating ‘me time’, also set them aside from the play sessions. Indeed, within mental health research more widely, there is some evidence – albeit limited – that relaxation techniques could support recovery from depression [44]. For the mothers, singing appeared to offer an immersive experience that provided some relief from the practical and emotional concerns of early motherhood. Importantly, the activity was also seen as a unique opportunity to engage in something designed for the mother rather than only for the baby, highlighting the mother’s own needs for care and nurture, a point also made by Feeley et al. [11]. Linked with this point is the value that the mothers placed on what they perceived as the authenticity of the singing activity, which moved away from more standard repertoire for mothers and babies (such as nursery rhymes) to include songs and music from around the world. This enabled mothers to contribute songs from their own cultures and backgrounds for the group to learn together, further emphasizing the feeling of community and group learning. This may be a key feature of the intervention, as it appeared to facilitate an emotional and social connection specifically with the music that allowed the mothers to relax and become absorbed in the activity with their baby, subsequently providing some relief from the symptoms of PND. Indeed, others have reported on the role of lullabies in enhancing feelings associated with motherhood and in understanding babies’ responses [45].

Finally, the enhanced mother-baby bond that some mothers reported as a result of singing is of note. It has been posited that singing may have developed out of ‘motherese’, a form of speech directed by mothers at their infants that consists of formalisations, repetitions, exaggerations and elaborations of ordinary adult vocal communication [46, 47]. Motherese is thought to be an interactive process between mother and baby [48], and singing may achieve similar responses from a baby such as enhanced engagement, visual attention and modulation of infant arousal [49, 50]. PND has been linked with reduced mother-infant bond [51], and it appears that singing may provide one mechanism for supporting this bond through the shared interaction of singing vocalisations. Indeed, singing with other populations has also been reported as a means of enhanced interpersonal communication and bonding [27–30]. Further, Mualem & Klein [52] demonstrated that musical interactions provided more opportunities for synchronisation between mothers and one-year olds, as well as positive emotional arousal, than play. It is possible that singing facilitates a unique mother-infant bonding experience, and further work is required in this area.

What, then, can we infer as to the specific benefits of community group singing for reducing symptoms of PND? Interestingly, the majority of the mechanisms distinct to singing were categorised under the ‘psycho-emotional mechanisms’ sub-theme. These mechanisms are concerned with the mother’s affective and psychological response to the singing activity, and may relate to subjective feelings, expression, action tendency or regulation invoked through the musical experience [53]. Music has for a long while been associated with strong emotional responses [54], and it could be argued that what we see in this group singing intervention is the use of singing to facilitate an emotional response to music that is context-specific to the experience of new motherhood. Indeed, Sloboda and Juslin [55] make clear that emotional responses to music occur in a complex interaction between the music, listener and situation, and are dependent on the goals and motives of the listener. While both singing and play elicited ‘feel good’ emotions such as happiness and a feeling of being uplifted, only singing appeared to elicit a more functional emotional response rooted in the needs of new motherhood: to have time to reframe the self, to feel immersed in an activity beyond looking after the baby alone, to feel competent as a mother and to feel bonded with the baby. The experience of functional positive emotions in relation to the experience of motherhood, facilitated through the creative act of singing, may help to explain the faster reduction in symptoms that this intervention elicits. Indeed, this response may also reflect the ability of the workshop leaders to recognise and respond to the women’s emotional state through the singing itself, modifying the songs to allow women to rest, be close to their baby or to learn new repertoire to take away from the session.

Finally, this work contributes to the wider body of literature pointing to the mental health benefits of singing. We saw in the opening of this article that singing can be life-enhancing [24], support recovery from serious or enduring mental illness [25] and provide a useful coping strategy in times of adversity [27]. Our findings confirm the recovery potential of signing, both in terms of supporting women to ‘feel good’ (Hedonia) and to ‘function well’ (Eudaimonia) [56]. Further, as discussed above, singing emerged as a tool to facilitate a feeling of closeness or bonding between mother and baby, which can be compromised when a women is experiencing symptoms of PND [51]. This supports Kreutz’s argument that singing may have emerged to enhance social bonding and mutual attachment [28] and echoes findings from our recent study demonstrating that singing, but not chatting, is associated with increases in maternal perception of emotional closeness with their baby [57]. Finally, our study resonates with the so-called ‘functional outcomes’ of singing identified in a previous study [26], with singing appearing to be a tool that can be modified to meet participants’ emotional needs in their particular context. Our findings make it clear that, for the women in this study, the impact of singing appeared to be *specific* to their needs as new mothers. Whether or not this specificity arose as a result of careful leadership from the facilitators and/or the innate qualities of singing itself requires further investigation. Indeed, this issue of specificity will be important to continue unpacking if we are to fully uncover the potential of singing to support diverse participant groups.

## Conclusions

This article uses a comparative qualitative methodology to describe the specific features of a community group singing intervention known to reduce moderate-severe symptoms of PND more rapidly than usual care: (1) the authentic, social and multicultural nature of the singing experience, which was not seen as ‘commercial’ and which drew upon global songs that were meaningful to the mothers as well as other creative forms; (2) the ability of singing to calm babies, both in and out of the sessions; (3) the singing session as ‘me time’ for mothers, that can be both relaxing and immersive; (4) the ability of singing to facilitate a sense of achievement and identity for mothers, particularly in relation to nurturing their babies and rediscovering a sense of self and purpose after the transition to motherhood; (5) singing as a means of enhancing the mother-baby bond. In summary, the psycho-emotional mechanisms of the activity emerge as central to the reported benefits, with community group singing appearing to facilitate the experience of functional positive emotions in relation to the experience of motherhood. Further research is needed to scrutinise the extendibility of this finding, as well as to further understand the complex interactions of responses to singing and recovery from symptoms of PND.

### Limitations

While one of the first to attempt to account for the mental health benefit of singing for new mothers, this article is not without its limitations. More women completed the singing intervention than the play intervention [38] and therefore more women were represented in the singing focus groups than the play. Although this means that the perspectives of fewer mothers are included in the play data, this is arguably a function of the singing intervention being the most effective for postnatal mothers. Additionally, not all mothers were able or willing to participate in the focus groups, potentially distorting the resulting data, and biasing them in favour of mothers who reported predominantly positive outcomes. Similarly, the focus groups were relatively short and therefore the richness of the data may be compromised. Further, the singing intervention was shown to be particularly effective for mothers with moderate-severe symptoms of PND [32], yet the focus groups were open to all mothers who participated in the activities, including those with milder symptoms. Indeed, the data are also limited by the characteristics of the sample, which includes a high percentage of degree-educated women in relatively high earning households. Further research will benefit from addressing these limitations in order to continue building the evidence base for the role of singing in maternal mental health.

## Abbreviations

EPDS: Edinburgh Postnatal Depression Scale
GP: General Practitioner
NHS: National Health Service
PND: Postnatal Depression
RCT: Randomised Controlled Trial
UK: United Kingdom
